# Supplementary material for: Complexome profiling of the Chlamydomonas psb28 mutant reveals TEF5 as an early PSII assembly factor
Source: Plant Cell. 2025 Mar 17;37(6):koaf055. doi: 10.1093/plcell/koaf055 (PMC12164590; doi:10.1093/plcell/koaf055)
Supplement: koaf055_Supplementary_Data [file koaf055_supplementary_data.zip › psb28_interactive.zip!/plots/intensity/qualityPlots/peptides/KIL8_Cre07.g326050.t1.1.html]

### Cre07.g326050.t1.1

Trivial name: KIL8  
SeqCoverage [median]: 1.10  
Mass: 211.36 kDa  
  
 

| Sequence | MissedCleavage | Mass [Da] | wtIntensity | muIntensity | Score |
| --- | --- | --- | --- | --- | --- |
| AEQLGLELEAAMTQRAK | 1 | 1857.96 | 260714.97 | 376666.74 | 79.34 |
| QVEQLR | 0 | 771.42 | 0.00 | 725.05 | 74.72 |
|  |  |  | 260715.0 | 377391.8 |  |
